# Therapeutic Inertia in Multiple Sclerosis Care: A Study of Canadian Neurologists

**DOI:** 10.3389/fneur.2018.00781

**Published:** 2018-09-24

**Authors:** Gustavo Saposnik, Xavier Montalban, Daniel Selchen, Maria A. Terzaghi, Fabien Bakdache, Alonso Montoya, Manuel Fruns, Fernando Caceres, Jiwon Oh

**Affiliations:** ^1^Division of Neurology, Department of Medicine, St. Michael's Hospital, University of Toronto, Toronto, ON, Canada; ^2^Laboratory for Social and Neural Systems Research, Department of Economics, University of Zurich, Zurich, Switzerland; ^3^Li Ka Shing Knowledge Institute, St. Michael's Hospital, University of Toronto, Toronto, ON, Canada; ^4^Department of Neurology-Neuroimmunology, Centre d'Esclerosi Múltiple de Catalunya (Cemcat), Hospital Universitari Vall d'Hebron, Barcelona, Spain; ^5^Medical Affairs, Neuroscience, Hoffmann-La Roche Limited, Mississauga, ON, Canada; ^6^Clinica Las Condes, Santiago, Chile; ^7^Instituto de Investigacion en Neurosciencias Buenos Aires, Buenos Aires, Argentina; ^8^Department of Neurology, Johns Hopkins University, Baltimore, MD, United States

**Keywords:** multiple sclerosis, disease-modifying therapy, therapeutic inertia, neuroeconomics, decision making, risk, Canada

## Abstract

**Introduction:** According to previous studies, therapeutic inertia (TI) may affect 7 out of 10 physicians who care for MS patients, particularly in countries where clinical guidelines are not widely used. Limited information is available on the prevalence of TI and its associated factors across Canada.

**Objectives:** (i) To evaluate factors associated with TI amongst neurologists caring for MS patients across Canada; (ii) to compare the prevalence of TI observed in Canadian neurologists to the prevalence of TI observed in Argentinean, Chilean, and Spanish neurologists (historical controls from prior studies).

**Design:** One hundred and eight neurologists with expertise in MS were invited to participate in an online study in Canada. Participants answered questions regarding their clinical practice, risk preferences, management of 10 simulated case-scenarios. The design of that study was similar to that of the prior studies completed in Argentina and Chile (*n* = 115). TI was defined as lack of treatment initiation or escalation when there was clear evidence of clinical and radiological disease activity (8 case-scenarios, 440 individual responses). A TI score was created & defined as the number of case-scenarios that fit the TI criteria over the total number of presented cases (score range from 0 to 8), with a higher score corresponding to a higher TI. TI scores observed in the Canadian study were compared with those observed in Argentina and Chile, as both studies followed the same design, case-scenarios and methodologies. Predictors of TI included demographic data, MS specialist vs. general neurologist, practice setting, years of practice, volume of MS patients and risk preferences.

**Results:** Fifty-five Canadian neurologists completed the study (completion rate: 50.9%). The mean age (±SD) was 38.3 (±15) years; 47.3% of the participants were female and 56.4% self-identified as MS specialists. Overall, 54 of 440 (12.3%) individual responses were classified as TI. 60% of participants displayed TI in at least one case-scenario. The mean TI score across Canada [0.98 (SD = 1.15)] was significantly lower than the TI score observed in the Argentinean-Chilean [1.82 (SD = 1.47); *p* < 0.001] study. The multivariable analysis revealed that older age (*p* = 0.018), years of experience (*p* = 0.04) and willingness to risk further disease progression by avoiding treatment initiation or treatment change (*p* = 0.043) were independent predictors of TI.

**Conclusions:** TI in Canada was observed in 6 out of 10 neurologists, affecting on average 1 in 8 therapeutic decisions in MS care. TI in Canada is significantly lower than in the other studied countries. Factors associated with TI include older age, lower years of experience, and willingness to risk disease progression by avoiding treatment initiation or treatment change. Differences in clinical practice patterns and adherence/access to accepted MS guidelines may explain how TI in Canada differs significantly from TI in Argentina-Chile.

## Background

Multiple sclerosis (MS) is (1) one of the most prevalent demyelinating conditions affecting nearly 100,000 people across Canada. On average, 8 women and 3 men are diagnosed with MS every day in Canada (2).

Therapeutic decisions in MS are cognitively demanding and a complex task partly because of the wide variety of available disease modifying agents that are each associated with different efficacy and safety profiles (3, 4). Furthermore, MS treatment options available in Canada have doubled in the last 10 years (5). Given the limited training that physicians are exposed to with respect to risk management and decision-making processes, in dynamic treatment landscapes, treatment decisions may be inadequate, leading to suboptimal patient care and overall poorer outcomes (6, 7). It can also be challenging for neurologists to balance the immediate management of treatment side effects (8) with the longer-term risks of MS disease progression and on the patient and on society (9). A more proactive management strategy, including earlier use of high-efficacy DMTs and close monitoring of the clinical and radiological response to treatment is recommended to slow the progression of physical and cognitive impairments in patients with relapsing-remitting multiple sclerosis (RRMS) (10–12). Treatment escalation has been shown to reducing relapse rates, disability progression, and MRI activity (13).

Therapeutic inertia (TI) is a term which was introduced in 2006 to define the absence of treatment initiation or intensification in patients when treatment goals are unmet (14–16). In the context of MS, TI is defined as the lack of treatment initiation or escalation when there is evidence of disease activity, based on clinical course and neuroimaging markers (3, 17, 18). TI usually affects 30–70% of clinicians caring for patients with chronic conditions (14–16). Physician factors (e.g., low tolerance to uncertainty, status quo bias) are considered to be the main contributors to TI, but remain poorly studied in MS (4, 19, 20).

We hypothesized that TI affects at least 50% of Canadian Neurologists but lower than in other countries, with personal attributes (e.g., age, risk preferences) influencing treatment decisions.

Accordingly, our goals were: (i) To evaluate factors associated with TI amongst neurologists caring for MS patients across Canada; (ii) to compare the prevalence of TI in neurologists in Canada to TI observed in neurologists from Argentina and Chile (historical controls from prior studies).

## Methods

### Study design and participants

We conducted a cross-sectional study using an online platform (Qualtrics.com). The study consisted of 10 MS case-vignettes and 2 behavioral experiments, which were administered to Canadian practicing neurologists between Dec 18, 2017 and May 4, 2018. Case-scenarios were designed by our research team and MS experts (GS, JO, DS, and XM). Overall, 8 cases were designed to assess appropriate escalation of treatment (where an absence of treatment change corresponds to TI), while the remaining 2 cases were designed to assess overtreatment (treatment change when there was no evidence of disease activity).

Behavioral experiments were designed to assess risk preferences in the health and financial domains as previously reported by our group (17, 20, 21). Specifically, participants were asked what would be the minimal payoff that they would consider over the equiprobable gamble of winning either 400 or 0 dollars (expected value of 200 dollars). The degree of risk aversion of each individual corresponded to the difference between the expected value of the risky option (200 dollars) and each participant's response (proxy of certainty equivalent) (17, 20).

A similar strategy was used to evaluate risk preference in the health domain. Participants were asked what minimum number of years of survival without treatment they would choose for their patient over a guaranteed 20-year survival treatment associated with a 20% probability of experiencing a side effect that may require hospitalization. The first choice reflects individuals who are averse to starting treatment, and are willing to accept a lower lifespan without treatment. Values lower than 10 healthy-years of survival would represent aversion to the risks of treatment, whereas higher values would represent risk-prone individuals. A study with a similar protocol and design was conducted in Argentina and Chile in 2017 (22). Details of the protocol were published in previous publications (17, 20).

#### Participants

Practicing neurologists actively involved in the care of patients with MS from across Canada were invited to participate in our study by the Canadian Consortium of MS clinics and Neuro-sens (Neuro-sens.com). Physicians whose practice was primarily in caring for MS patients were classified as “MS specialists.” All participants received compensation for completing the survey.

#### Definitions

For the primary analysis, we used an accepted definition of disease activity that would prompt to treatment initiation or escalation (3, 23, 24). Disease activity was defined as the presence of a clinical relapse plus the presence of more than four new brain lesions in follow-up magnetic resonance imaging (MRI) scans or at least one gadolinium-enhancing lesion (23, 24). The use of these definitions combining a clinical relapse and MRI activity is consistent with recent evidence regarding the risk of treatment failure among patients receiving interferon-β (25). Disease progression was defined as at least one point worsening from baseline in the Expanded Disability Status Scale (EDSS) score (26).

Recent meta-analysis confirmed that alemtuzumab, natalizumab, and fingolimod are the best available choices for preventing clinical relapses in patients with RRMS (27). The current landscape of DMTs for the treatment of RRMS includes first-line therapies (beta interferons, glatiramer acetate, teriflunomide, and dimethyl fumarate) and second-line therapies (fingolimod, natalizumab, alemtuzumab, etc.) as recently summarized in a consensus algorithm (3). For the present analysis, we used the aforementioned scheme according to the current clinical practice (3, 18). Data from historical controls were obtained from previous/in press publications (22).

#### Outcome measures

The primary outcome of the study was the proportion of participants who exhibited TI.

TI was determined as a score and as a categorical variable. We created a TI score to represent the number of case scenarios where treatment initiation or escalation was warranted (numerator) divided by the total number of case-scenarios that measured TI (denominator; *n* = 8). TI as a categorical variable (presence/absence) was determined as the lack treatment initiation or escalation given disease activity in at least one case scenario.

Secondary outcome measures included the association between clinical variables and risk preference with TI score.

### Statistical analysis

The primary analysis was a descriptive assessment of the presence of TI (categorical) and TI score. We then compared the TI score obtained from Canadian participants with the TI scores obtained from other countries (given the identical case-scenarios, definitions, and methodologies) using a Fisher's exact test. Risk assessment in the health domain was assessed using the median split. A multivariate regression analysis was completed to determine the association between physicians' characteristics with the primary outcome of interest (TI score). We included the following explanatory variables: age, gender, MS patients seen per week, practice setting (academic vs. non-academic), proportion of time devoted to clinical care, co-author in a peer reviewed publication within the last 12 months (yes/no), and risk preferences. All tests were 2-tailed, and *p* < 0.05 were considered significant. We used STATA 13 (College Station, TX: StataCorp LP) to conduct all analyses.

The study was approved by the Research Ethics Board of St. Michael's Hospital, University of Toronto, Canada.

Online informed consent was obtained from all participants.

## Results

Of the 108 neurologists from across Canada who were invited to participate in the study, 78 cooperated (cooperation rate: 72.2%) and 55 (completion rate: 50.9%) completed the study. We found no significant difference in age, sex, and years of experience between participants who completed and those who did not complete the study.

Overall, the mean age (SD) was 38.3 (±14.9) years; 26 (47.3%) were female. Thirty-one participants (56.4%) primarily focused their practice on MS care. On average, participants had 13 (±11.2) years of experience and assessed 22.2 (±14.6) MS patients per week. Table 1 summarizes baseline characteristics of the study population.

**Table 1:** Baseline characteristics of participants.

**Characteristics**	**Total (%) *n* = 55**
**Age** (mean ± SD), in years	38.3 ± 14.9
**Sex**
Female	26 (47.3)
**Specialty**
MS specialists	31 (56.4)
General Neurologists who care for MS patients	24 (43.6)
**Practice setting**
Academic	41 (74.6)
Community	14 (24.4)
**% time in clinical practice**	
50–74% of their time	22 (40.0)
>75%	30 (54.6)
**Years in practice**, mean (±SD)	13.3 ±11.2
**MS patients seen per week**, mean (±SD)	22.2 ± 14.6
**Author of a peer-reviewed publication in the last 12 months**	31 (56.4)

*Numbers in brackets indicate percentages, unless otherwise indicated*.

TI was present in 60.0% of participants in at least one case scenario. Thirteen participants (23.6%) showed TI in two or more case-scenarios. The mean TI score was 0.98 (±1.15). The analysis of individual responses revealed that TI was present in 12.3% of participants' responses (54/440).

For the risk assessment, the median value was 200 dollars (interquartile range 200–220) of safe earnings (instead of the 50/50 gamble of winning $400/$0), and 15 years (interquartile range 15–16) of survival without treatment. Participants who chose over 15 healthy years of survival without treatment (instead of 20 years with a 20% probability of side effects) had higher TI scores (mean TI score 1.47 vs. 0.73; *p* = 0.03). There were no differences in the TI score by risk assessment in the financial domain (mean TI score 1.06 vs. 0.95; *p* = 0.74).

**Figure 1:**
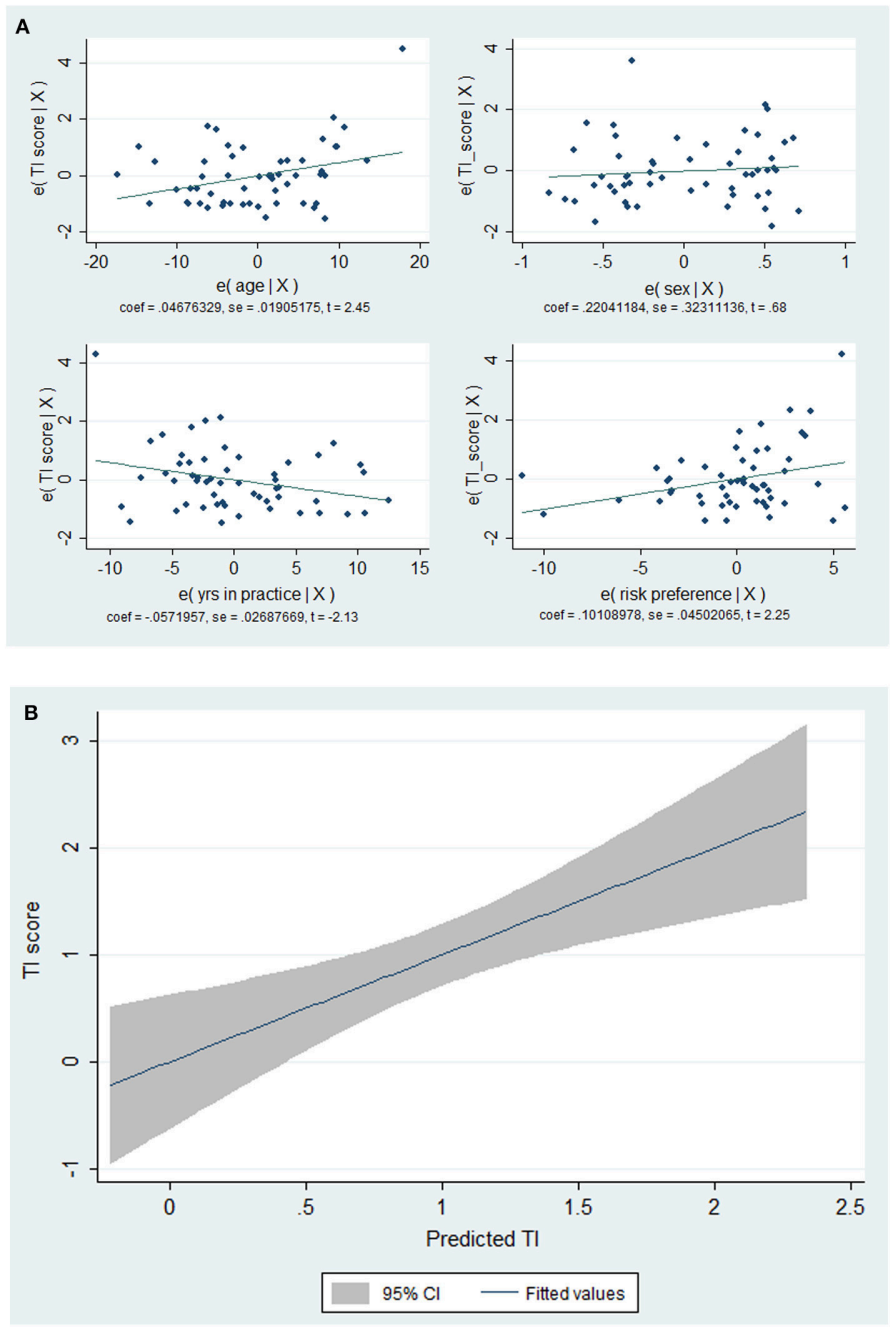
Plots between factors associated TI scores **(A)** and TI score vs. predicted values by the model **(B)**. Note the direct relationship between age and propensity to take risks (by selecting the no treatment option) in the health domain and TI scores. Conversely, the lower the clinical experience (years of practice) the higher the TI scores **(A)**. The fitted model showed a linear relationship between TI scores and the predicted values **(B)**.

The multivariate analysis revealed that older age (β 0.05, 95%CI 0.008–0.085; *p* = 0.018), less years of experience (β -0.06, 95%CI -0.11 to -0.03; *p* = 0.04) and inclination to opt for no treatment change when there is a risk of disease progression (β 0.10, 95%CI 0.01–0.19; *p* = 0.043) were independent predictors of TI (Figure 1).

### TI score in canada compared with historical controls

The TI score among Canadian participants was significantly lower than the TI scores of historical controls. For instance, the TI score in the Argentinian-Chilean cohort (*n* = 115) was 1.82 ± 1.47 (*p* ≤ 0.001).

## Discussion

TI is a common phenomenon in the management of MS patients (4). In the present study comprising neurologists with expertise in MS care from across Canada, TI was observed in 6 out of 10 participants. On average, for every 8 case-scenarios that warranted treatment escalation (or initiation), TI was demonstrated in one case. Factors most relevant to demonstrating TI were older age, lower years of experience, and willingness to accept risks of disease progression by avoiding treatment change in spite of the availability of more effective treatments.

Of interest is the comparison of TI in neurologists from Canada vs. that of neurologists from other countries (Argentina, Chile, and Spain) (22). Our analysis revealed that Canadian participants had a significantly lower TI. The underlying causes are unknown and warrant further studies. Cultural and practice-based differences, adherence to MS guidelines, and policy funding for DMTs may potentially explain our findings.

Our study has some limitations. First, the sample size is small, but representative of prescribers of MS agents from across Canada. Second, some participants' responses may reflect local administrative or health policy limitations in the prescription of disease modifying agents. Third, we were not able to compare baseline differences among countries to identify potential explanatory factors influencing TI.

Despite these limitations, our study suggests that TI affects at least 6 out of 10 neurologists caring for MS patients and is observed as frequently as 1 in 8 case-presentations when evidence of disease activity warranting treatment intensification. These findings, if confirmed in larger studies, have significant implications from a clinical care perspective, and suggest that educational interventions targeting the identified factors that influence TI may be warranted.

Future directions would include a larger study including several countries to identify factors and health system differences influencing TI. Moreover, future studies should also identify how neurologists weigh different factors (e.g., years since the MS diagnosis, number and severity of relapses, MRI findings, patients' preferences etc.) when making therapeutic decisions. Such information could help improve our understanding of clinical decision making in MS and may inform educational interventions that can ultimately lead to better outcomes in MS care.

## Disclosure

GS is supported by the Heart and Stroke Foundation Career Scientist Awards following an open peer-reviewed competition. JO has received research funding from the MS Society of Canada, National MS Society, Brain Canada, and Biogen-Idec. JO has received personal compensation for consulting or speaking from EMD-Serono, Sanofi-Genzyme, Biogen-Idec, Roche, and Novartis.

## Author contributions

GS: study concept and design, creation of the educational intervention, acquisition of data, analysis and interpretation of the data, and obtaining funding. MT: study implementation, interpretation of the data, and critical revision of the manuscript for intellectual content. FB, DS, and AM: interpretation of the data, critical revision of the manuscript for intellectual content. FC and MF: critical revision of the manuscript for intellectual content. XM and JO: supervision of MS case-scenarios, interpretation of the data, critical revision of the manuscript for intellectual content.

### Conflict of interest statement

The authors declare that the research was conducted in the absence of any commercial or financial relationships that could be construed as a potential conflict of interest.
